# WenTong HuoXue Cream Can Inhibit the Reduction of the Pain-Related Molecule PLC-*β*3 in the Dorsal Root Ganglion of a Rat Model of Diabetic Peripheral Neuropathy

**DOI:** 10.1155/2018/3416936

**Published:** 2018-02-11

**Authors:** Chengcheng Feng, Lijuan Xu, Shiyun Guo, Qian Chen, Yuguo Shen, Deng Zang, Li Ma

**Affiliations:** ^1^Traditional Chinese Medicine Hospital, Xinjiang Medical University, Ürümqi, China; ^2^Xinjiang Medical University, Ürümqi, China

## Abstract

WenTong HuoXue Cream (WTHX-Cream) has been shown to effectively alleviate clinical symptoms of diabetic peripheral neuropathy (DPN). This study investigated the gene and protein expression of the pain-related molecule PLC-*β*3 in the dorsal root ganglion (DRG) of DPN rats. 88 specific pathogen-free male Wistar rats were randomly divided into placebo (10 rats) and DPN model (78 rats) groups, and the 78 model rats were used to establish the DPN model by intraperitoneal injection of streptozotocin and were then fed a high-fat diet for 8 weeks. These rats were randomly divided into the model group, the high-, medium-, and low-dose WTHX-Cream + metformin groups, the metformin group, the capsaicin cream group, and the capsaicin cream + metformin group. After 4 weeks of continuous drug administration, the blood glucose, body weight, behavioral indexes, and sciatic nerve conduction velocity were measured. The pathological structure of the DRG and the sciatic nerve were observed. PLC-*β*3 mRNA and protein levels in the DRG of rats were measured. Compared with the model group, the high-dose WTHX-Cream group showed increased sciatic nerve conduction velocity, improved sciatic nerve morphological changes, and increased expression of PLC-*β*3 mRNA and protein in the DRG. This study showed that WTHX-Cream improves hyperalgesia symptoms of DPN by inhibiting the reduction of PLC-*β*3 mRNA and protein expression in the diabetic DRG of DPN rats.

## 1. Introduction

Diabetic peripheral neuropathy (DPN) is a common chronic complication of diabetes and involves distal symmetrical neuropathy (major manifestation), numbness, pain, hyperalgesia, and myasthenia. The neuropathic pain generated by DPN severely impacts the quality of life of diabetic patients [1]. Currently, DPN treatment includes improving microcirculation, promoting neurotrophy, and relieving pain [2]. However, due to the multifactor interactive pathogenesis of DPN, clinical drugs targeting a single pathway have been shown to have poor curative effects [3]. Traditional Chinese medicine has the advantages of multiple components and multiple targets and thus may be valuable in treating DPN. WTHX-Cream (a local heating cream for improving blood perfusion) is produced based on the theory of traditional Chinese medicine and consists of seven traditional Chinese herbs: Chuanxiong, safflower, Ramulus Cinnamomi,* Asarum*,* Angelica*,* Allolobophora caliginosa* trapezoids, and pepper; this treatment is believed to promote blood circulation and eliminate stasis. External use of this cream on the affected region is believed to activate the blood, resolve stasis, and dredge collaterals to relieve pain. Our previous study showed that WTHX-Cream can effectively relieve the symptoms of DPN and reduce allergic pain [4].

Pain can be divided into nociceptive pain and neuropathic pain (or mixed pain of the two types of pain). DPN is classified as neuropathic pain, and its pathological mechanism is complex; phospholipase C-*β*3 (PLC-*β*3) plays an important role in pain occurrence. PLC-*β*3 belongs to the phospholipase molecule C family, which contains a variety of members, including PLC-*β*, -*γ*, -*δ*, and -*ε*. PLC-*β* includes PLC-*β*1, PLC-*β*2, PLC-*β*3, and PLC-*β*4. PLC-*β*1, PLC-*β*3, and PLC-*β*4 are distributed in the dorsal root ganglion (DRG), and PLC-*β*3 has the highest transcription levels [5]. Many studies have confirmed that PLC-*β*3 is associated with pain. Related studies have shown that, in spared nerve injury, the PLC-*β*3 content in the DRG of the same side was significantly decreased but did not change on the noninjury side, indicating that the PLC-*β*3 level of the DRG after peripheral nerve injury is reduced [6].

In combination with analysis of the pain perception transmission mechanism, histopathology, real-time quantitative PCR, and immunoblotting were used to explore the effects of WTHX-Cream on DPN based on histopathological changes and pain-related molecule PLC-*β*3 mRNA and protein level changes. This study aimed to provide a theoretical basis and guidance for the clinical treatment of DPN with WTHX-Cream and to develop new ideas for the external use of Chinese traditional medicine in the treatment of DPN.

## 2. Materials and Methods

### 2.1. Animals

88 specific pathogen-free male Wistar rats weighing 80–130 g were purchased from Xinjiang Experimental Animal Research Center (production license number: SCXK (new) 2011-0001; animal certificate number: 65000200000408). Rats were fed separately, with 5 rats in each cage. Humidity was controlled within the range of 30%–50%, and temperature was controlled within 20–26°C. Rats were fed for 1 week under natural light, with freely available food and drinking water, and they were randomly divided into the placebo (10 rats) and DPN model (78 rats) groups according to the random number table method. Placebo cream was applied to rats in the placebo group, and they were given distilled water via gavage. The remaining rats in the DPN model group were injected with streptozotocin (STZ) and were fed a high-fat diet for 8 weeks to develop the DPN model. The DPN model groups were subdivided into the model group (placebo cream + lavage with distilled water, 11 rats), high-dose WTHX-Cream + metformin group (high-dose WTHX-Cream + lavage with metformin, 11 rats), medium-dose WTHX-Cream + metformin group (medium-dose WTHX-Cream + lavage with metformin, 11 rats), low-dose WTHX-Cream + metformin group (low-dose WTHX-Cream + lavage with metformin, 11 rats), metformin group (placebo cream + lavage with metformin, 10 rats), capsaicin group (capsaicin cream + lavage with distilled water, 10 rats), and capsaicin cream + metformin group (capsaicin cream + lavage with metformin, 11 rats).

### 2.2. Drug

WTHX-Cream is a Chinese drug preparation containing Chuanxiong, safflower, Ramulus Cinnamomi,* Asarum*,* Angelica*,* Allolobophora caliginosa* trapezoids, and pepper. The preparation was generated as follows: after the above seven Chinese drugs were washed, they were crushed into coarse particles; then 70% ethanol was added 8 times; the drugs were extracted twice (2 h each time) and they were concentrated into a liquid with a relative density of approximately 1.05 by exposure to low temperature and decompression; then they were added to the selected cream substrate [7]. The cream substrate included stearic acid, glyceryl monostearate, Azone, and hydroxyphenethyl ester and was provided by the Traditional Chinese Medicine Hospital affiliated with Xinjiang Medical University (batch number 20151115); the capsaicin cream contained capsaicin (0.75 mg per gram), which was purchased from Guizhou Green Sun Pharmaceutical Co., Ltd. (batch number 20150601). Metformin hydrochloride tablets were purchased from Beijing Zhonghui Pharmaceutical Co., Ltd. (batch number CO120150407). The placebo cream was the basic substance used for the preparation of WTHX-Cream and was provided by the Traditional Chinese Medicine Hospital affiliated to Xinjiang Medical University.

### 2.3. Modeling, Grouping, and Administration

According to previous research methods [8, 9], Wistar male rats were adaptively fed for one week and were then randomly divided into two groups according to body weight. Ten rats fed with basic feedstuff were used as the placebo group; the remaining rats were used as DPN model groups, and these rats were fed a high-sugar and high-fat diet (70% conventional feed, 20% sucrose, and 10% lard) for 6 weeks, fasted for 12 h, and given 35 mg/kg of low-dose STZ (dissolved into 0.1 mol/L citrate buffer, pH 4.2). The control group was given an equal volume of 1% sodium citrate buffer. After 7 days, blood glucose was measured via tail bleeds. Fasting blood glucose ≥ 11.1 mmol/L was used as the standard for diabetic rats. The modeled diabetic rats were fed a high-glucose, high-fat diet for 8 weeks to induce DPN. The DPN rat model was established as follows: the sensory and motor conduction velocity of the sciatic nerve of the lower limb of diabetic rats was measured by electromyography. The DPN rat model was successfully established when the sensory or motor conduction velocity was slowed by more than 11%. Drug administration was performed following the method used by Leng et al. [10]: the placebo group was given placebo cream (0.8 g per rat), and rats were lavaged with distilled water at 10 ml/kg; the model group was given placebo cream (0.8 g per rat) and was lavaged with distilled water at 10 ml/kg; the high-, medium-, and low-dose WTHX-Cream + metformin groups were coated with WTHX-Cream (0.8 g per rat, 0.4 g per rat, and 0.2 g per rat, resp.) and were lavaged with metformin at 0.18 g/kg; the metformin group was coated with placebo cream (0.8 g per rat) and lavaged with metformin at 0.18 g/kg; the capsaicin cream group was coated with capsaicin cream, 0.2 g per rat, and was lavaged with distilled water at 10 ml/kg; and rats in the capsaicin cream + metformin group were coated with capsaicin cream, 0.2 g per rat, and were lavaged with metformin at 0.18 g/kg. The hind limbs of all rats were shaved and were coated with cream after 24 h. Then, the heads of the rats were fixed with a connector. After 4 h, the test drug was washed off with warm water. Drugs were continuously administered for 4 weeks, once per day.

### 2.4. Determination of Nerve Conduction Velocity

#### 2.4.1. Motor Nerve Conduction Velocity (MNCV) Determination

Rats were anesthetized and were fixed in a prone position. A stimulating needle electrode was placed at the efferent location of the sciatic nerve on the incisura ischiadica, and the needle electrode was used to record the passing site of the sciatic nerve on the ankle joint. The reference electrode was located at a 1 cm distance between the stimulus electrode and recording electrode. With single pulse square wave stimulation, 5 volts of constant voltage was used, the current size was 20–40 mA, the wave width was 0.1 ms, the stimulus intensity was 1.5 times the threshold, and time from the stimulation of the nerve to the occurrence of the distal muscle action potential was the latency. The distance between the stimulus electrode and recording electrode was accurately measured on the animal surface. The MNCV from the ischiadic tuberosity to the ankle joint was calculated with the following formula: the distance between the stimulus electrode and the recording electrode/latency.

#### 2.4.2. Sensory Nerve Conduction Velocity (SNCV) Determination

The sensory nerve stimulation point was located at the distal end of the tail, the recording electrode was located at the proximal end of the tail, and distance between the recording electrode and stimulation point was approximately 2-3 cm. The amount of stimulation was gradually increased. The sensory nerve conduction velocity (m/s) = the distance conducted by the composite action potential/the latency of the compound action potential.

### 2.5. Behavioral Observation

#### 2.5.1. Thermal Hyperalgesia Test

In a quiet environment, rats were placed in the observation box for 15 min to adapt to the environment before determination. The light illuminates in the lateral metapedes footplate skin. The time from irradiation to the foot reaction, which was defined as the latent period, was measured, and the radiation treatment was repeated 3 times at 5-minute intervals. The longest irradiation time was 30 s to avoid damage to rats by long-term exposure to irradiation. Meanwhile, the average value was recorded.

#### 2.5.2. Cold Allodynia Test

After rat fixation, 0.1 ml of acetone was gently dropped on the lateral planta of the hind limbs, and the heat dissipated by acetone was used to induce cold allodynia in rats. The test was repeated 3 times with an interval of 5 min. Meanwhile, the average time for foot withdrawal was recorded, and the shortest time was 0.5 s.

#### 2.5.3. Mechanical Hyperalgesia Test

After rat fixation, a safety pin was used to appropriately puncture the lateral planta. The test was repeated 3 times with an interval of 5 min. Meanwhile, the average time for foot withdrawal was recorded, and the shortest time was 0.5 s.

#### 2.5.4. Mechanical Pain Threshold Measurement

Rats were placed in the observation box for 15 min to adapt to the environment before determination. Then, 0.4 g of von Frey fiber filament was used to vertically stimulate the middle planta to observe the foot withdrawal response. The occurrence of lifting the foot or licking foot behavior was defined as a positive reaction, and the absence of these behaviors was defined as a negative reaction. Each animal was stimulated 10 times, and the tests were conducted with an interval of 30 s. The percentage of the foot withdrawal response = the number of foot withdrawals/10 × 100%.

### 2.6. Histopathology of DRG Tissue and an Ultrastructural Observation of the Ischiadic Nerve

After drug administration, 0.5 ml/100 g (body weight) of 20% urethane was used to anesthetize rats through intraperitoneal injection, blood was drawn from the abdominal aorta, and then L4/L5 DRG tissue and part of the sciatic nerve were collected. The tissues were then fixed with 10% formaldehyde, stained with HE and toluidine blue, and histopathologically examined via light microscopy. At the same time, part of the sciatic nerve was fixed with glutaraldehyde, and thin sections were generated for an electron microscopic evaluation of the ultramicrostructure.

### 2.7. PLC-*β*3 Determination of the DRG

After drug administration, anesthesia with 10% chloral hydrate (350 mg/kg) was performed, and L4/L5 dorsal root nerve tissue was quickly removed. PLC-*β*3 mRNA expression was detected by fluorescence quantitative qPCR amplification (SYBR Green dye assay, *β*-actin as an internal reference) after extraction of total RNA and determination of RNA purity by electrophoresis. Fluorescent quantitative PCR primers were designed against the PLC-*β*3 sequence in the NCBI database by Primer-BLAST software and were synthesized by Urumqi Kuntairui Biotechnology Co., Ltd. The primer sequences are shown in Table 1. Total protein was rapidly extracted from the remaining tissue with liquid nitrogen for Western blot analysis. Semidry electrophoresis gels were transferred to PVDF membranes and then placed in confining liquid containing 5% skim milk powder for 1 h. Thereafter, a rabbit anti-PLC-*β*3 antibody (dilution ratio 1 : 1000, purchased from Abnova, batch number PAB17281) was added, gently stirred, and incubated at 4°C overnight. Then, the corresponding secondary antibody (G-anti-r-HRP) was applied to the blot, and the blot was incubated at room temperature for 2 h, followed by chemiluminescence development.

### 2.8. Statistical Methods

SPSS 19.0 statistical software was used. Multiple-group comparisons were performed by one-way ANOVA. The LSD method and Dunnett's T3 method were used for pairwise multiple comparisons. Data are expressed as the mean ± SD; *P* < 0.05 was considered significant. GraphPad Prism 5.0 was used to assist with mapping.

## 3. Results

### 3.1. Establishment of the DPN Model

**Figure 1:**
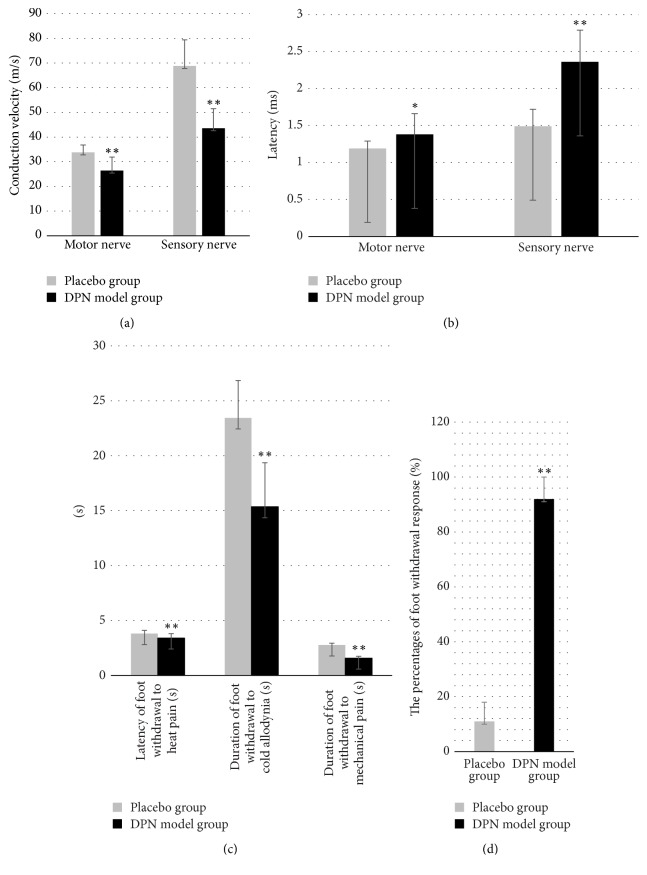
Analysis of (a) nerve conduction velocity, (b) nerve conduction latency, and ((c) and (d)) behavioral indicators in rats. All values given are the mean ± SD. ^*∗∗*^*P* < 0.01 and ^*∗*^*P* < 0.05 versus the placebo group.

During the model establishment period, rats in the placebo group were in good mental condition, and their weight continued to increase. Rats in the DPN model group lost weight (*P* < 0.01), while rats in the DPN model group displayed polyuria, a yellow coat color, and slow movement. During the modeling period, some rats had fundus lesions, which showed white turbidity. Electromyography was used to measure the motor neuron and sensory nerve conduction latencies, and the MNCV and SNCV were calculated before and after modeling. The motor nerve and sensory nerve conduction latencies were prolonged in the DPN model groups, and MNCV and SNCV were slower in the DPN model group than in the placebo group (*P* < 0.01). The results indicated successful establishment of the DPN rat model (Figures 1(a) and 1(b)). Measurement of the rat behavioral index before drug administration showed that the latency of foot withdrawal to heat pain, duration of foot withdrawal to cold allodynia, and duration of foot withdrawal to mechanical pain were shortened and that the percentages of foot withdrawal response were increased (*P* < 0.01), indicating that the pain thresholds for cold allodynia, heat pain, and mechanical pain were reduced (Figures 1(c) and 1(d)). After the data were measured, three rats died in DPN model group before the group was given the drug. During drug administration, some rats died because of high blood sugar, including one that died in the placebo group, three that died in the model group, one that died in the high-dose WTHX-Cream and metformin group, two that died in the middle-dose WTHX-Cream and metformin group, and two that died in the capsaicin cream + metformin group. Hence, after the end of the experiment, a total of 76 rats were statistically analyzed.

### 3.2. Effect of WTHX-Cream on the Body Weight and Blood Glucose of DPN Rats

**Figure 2:**
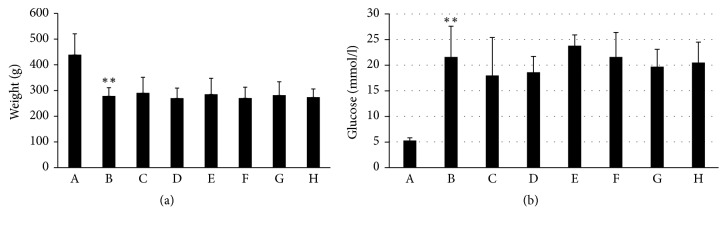
Effect of WTHX-Cream on the body weight and blood glucose of DPN rats. (a) Body weight; (b) blood glucose. All values given are the mean ± SD. ^*∗∗*^*P* < 0.01 versus the placebo group. A: placebo group; B: model group; C: high-dose WTHX-Cream + metformin group; D: medium-dose WTHX-Cream + metformin group; E: low-dose WTHX-Cream + metformin group; F: metformin group; G: capsaicin group; H: capsaicin cream + metformin group.

After 4 weeks of drug administration, the differences in body weight and blood glucose were significantly different between the DPN model group and placebo group. There were no significant differences between each administration group and the model group or among the administration groups (Figures 2(a) and 2(b)).

### 3.3. WTHX-Cream Can Improve the Pain Threshold of DPN Rats

**Figure 3:**
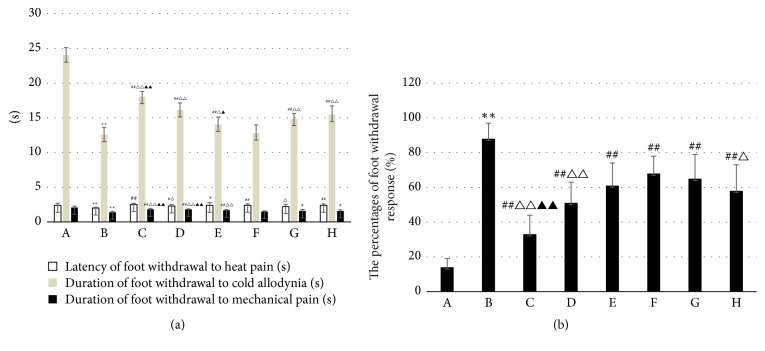
Effect of WTHX-Cream on the behavioral indicators in rats. (a) Latency of foot withdrawal to heat pain and duration of foot withdrawal to cold allodynia and mechanical pain; (b) the percentages of foot withdrawal response; all values given are the mean ± SD. ^*∗∗*^*P* < 0.01 versus the placebo group; ^#^*P* < 0.05 and ^##^*P* < 0.01 versus the model group; ^△^*P* < 0.05 and ^△△^*P* < 0.01 versus the metformin group; ^▲^*P* < 0.05 and ^▲▲^*P* < 0.01 versus the capsaicin cream + metformin group. A: placebo group; B: model group; C: high-dose WTHX-Cream + metformin group; D: medium-dose WTHX-Cream + metformin group; E: low-dose WTHX-Cream + metformin group; F: metformin group; G: capsaicin group; H: capsaicin cream + metformin group.

Compared with the model group, the drug administration groups showed improved indexes. Duration of foot withdrawal to cold allodynia and duration of foot withdrawal to mechanical pain were higher in the high-dose WTHX-Cream + metformin group than in the metformin group and capsaicin cream + metformin group (*P* < 0.01). The percentages of the foot withdrawal response were lower than those of the metformin group and capsaicin cream + metformin group (*P* < 0.01) (Figures 3(a) and 3(b)).

### 3.4. WTHX-Cream Can Improve Sciatic Nerve Conduction in Rats

**Figure 4:**
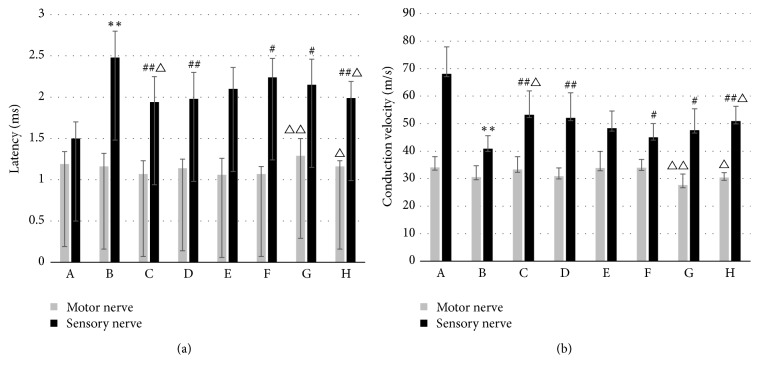
Effect of WTHX-Cream on the nerve conduction velocity in DPN rats. (a) Nerve conduction latency; (b) nerve conduction velocity; all values given are the mean ± SD. ^*∗∗*^*P* < 0.01 versus the placebo group; ^#^*P* < 0.05 and ^##^*P* < 0.01 versus the model group; ^△^*P* < 0.05 and ^△△^*P* < 0.01 versus the metformin group. A: placebo group; B: model group; C: high-dose WTHX-Cream + metformin group; D: medium-dose WTHX-Cream + metformin group; E: low-dose WTHX-Cream + metformin group; F: metformin group; G: capsaicin group; H: capsaicin cream + metformin group.

In the drug administration groups, the MNCV and latency were not significantly improved, but the sensory nerve conduction effect was more pronounced. Compared with the placebo group, the model group showed a prolonged latent period of sensory nerve conduction (*P* < 0.01). Compared with the model group, the drug administration groups showed a shortened sensory nerve latency and accelerated conduction velocity (*P* < 0.01), which were more pronounced in the high-dose WTHX-Cream + metformin group and the capsaicin cream + metformin group (*P* < 0.01) (Figures 4(a) and 4(b)).

### 3.5. Effects of WTHX-Cream on the Pathological Structure of the DRG and Ultrastructural Changes of the Sciatic Nerve in Rats

**Figure 5:**
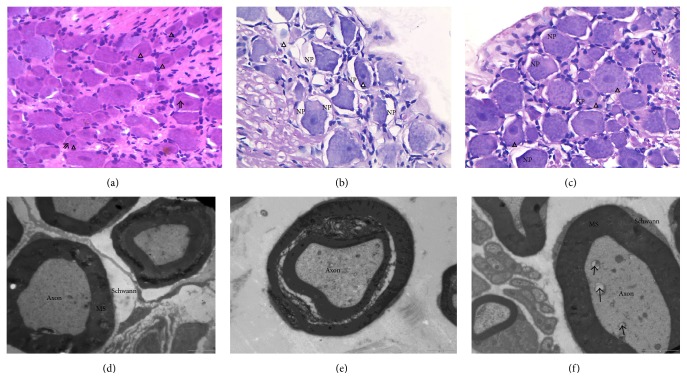
Effect of WTHX-Cream on the pathology of the DRG and ultrastructure of the sciatic nerve in DPN rats. Light microscopy: (a) placebo group, (b) model group, and (c) high-dose WTHX-Cream + metformin group. Electron microscopy: (d) placebo group, (e) model group, and (f) high-dose WTHX-Cream + metformin group. *⇑*: Nissl body (light microscopy); NP: neuronal pool; △: nucleus; MS: myelin; ↑: vacuolization (electron microscopy); Schwann: Schwann cells.

In the placebo group, light microscopy indicated that the neuronal cell structures of the DRG were intact, cytoplasm was rich, nuclei were round or oval and were located in the middle of the cell body, and Nissl bodies were clearly observed in the cytoplasm. Electron microscopy showed dense and uniform myelinated nerve fibers, concentrically arranged myelinated lamellae, axons without swelling and atrophy, neatly arranged axonal nerve fibers and microtubules, and normal Schwann cells (Figures 5(a) and 5(d)). In the model group, light microscopy indicated that neuronal cell structures of the DRG were degenerated, most of the cell bodies were atrophied, the neuron pool was broadened, Nissl bodies within the cytoplasm were dissolved and had an unclear structure, and some nuclei had dissolved and disappeared. Electron microscopy showed widened myelin in myelinated nerve fibers, a loose structure, disorderly arrangement, severe lamellar structure damage and lamellar isolation, severe nerve demyelination, decreased axonal density, and obvious vacuolar degeneration (Figures 5(b) and 5(e)). Both the metformin group and capsaicin group showed the same morphological and ultrastructural changes as the model group. In the high-dose WTHX-Cream + metformin group, light microscopy indicated that the neuronal cell structures of the DRG were partially degenerated and atrophied, the neuron pool was slightly widened, there was partial dissolution or disappearance within the cytoplasm, and individual cells were fragmented or disappeared. Electron microscopy showed that the myelinated myelin sheath layer structure was basically normal, the overall vacuolar degeneration was significantly improved compared with that of the capsaicin cream + metformin group, only mild myelofibrosis was observed, and the axonal lesions were not obvious (Figures 5(c) and 5(f)). Under light microscopy and electron microscopy, the pathological changes of the medium- and low-dose WTHX-Cream + metformin groups and capsaicin cream + metformin group were between those of the model group and the high-dose WTHX-Cream + metformin group. The neurons of the DRG were partially degenerated and atrophied, the neuronal pool was slightly widened, Nissl bodies were dissolved in the cytoplasm or had disappeared, and part of the nucleus was fragmented or had disappeared. Electron microscopy showed swelled myelination, demyelination in some nerves, varying degrees of axonal degeneration, normal nerve fiber filament structure, and partial occurrence of vacuolar degeneration.

### 3.6. WTHX-Cream Can Increase PLC-*β* Expression in the DRG of Rats

**Figure 6:**
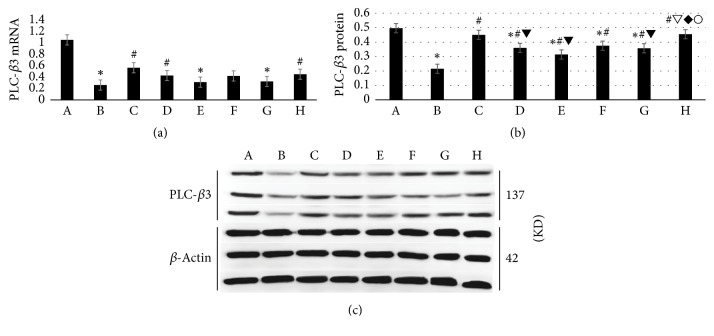
WTHX-Cream can increase PLC-*β* expression in the DRG of rats. (a) PLC-*β*3 mRNA; ((b) and (c)) PLC-*β*3 protein. All values given are the mean ± SD. ^*∗*^*P* < 0.05 versus the placebo group; ^#^*P* < 0.05 versus the model group; ^▼^*P* < 0.05 versus the high-dose WTHX-Cream + metformin group; ^▽^*P* < 0.05 versus the middle-dose WTHX-Cream + metformin group; ^*◆*^*P* < 0.05 versus the low-dose WTHX-Cream + metformin group; ^○^*P* < 0.05 versus the capsaicin group. A: placebo group; B: model group; C: high-dose WTHX-Cream + metformin group; D: medium-dose WTHX-Cream + metformin group; E: low-dose WTHX-Cream + metformin group; F: metformin group; G: capsaicin group; H: capsaicin cream + metformin group.

The relative expression of PLC-*β*3 mRNA in the model group was decreased compared with that of the placebo group (*P* < 0.05); the relative PLC-*β*3 mRNA levels in the high-dose WTHX-Cream + metformin group, medium-dose WTHX-Cream + metformin group, and capsaicin + metformin group were increased compared with that of the placebo group (*P* < 0.05), and there was no significant difference among the three groups (Figure 6(a)). The Western blot results showed that the expression of the PLC-*β*3 protein in the DRG in the model group was lower than that in the placebo group (*P* < 0.05). PLC-*β*3 protein expression in the DRG in the drug administration group was increased compared with that in the model group (*P* < 0.05). The PLC-*β*3 protein level was higher in the high-dose WTHX-Cream + metformin group than in the middle-dose WTHX-Cream + metformin group or the low-dose WTHX-Cream + metformin group (*P* < 0.05), but there was no significant difference between the high-dose WTHX-Cream + metformin group and the capsaicin + metformin group (Figures 6(b) and 6(c)).

## 4. Discussion

DPN is one of the most common chronic complications of diabetes mellitus and is a major risk factor for foot ulcers, infections, and gangrene. The major clinical symptoms are hyperalgesia and sensory disturbances. Because of its complicated pathogenesis, clinical treatment with drugs is limited. In traditional Chinese medicine, DPN belongs to the “arthralgia” category, which includes clinical symptoms such as numbness, pain, and burning and formication sensations. Traditional Chinese medicine defines the mechanism of DPN as follows: for patients suffering from long-term diabetes, blood and qi within the viscera and bowel are disharmonious, and pathological products, such as phlegm, toxin, and static blood, are blocked in blood vessels and meridians, which will not nourish limbs and eventually produce pain and even numbness [11, 12]. WTHX-Cream can activate blood, resolve stasis, and dredge collaterals to relieve pain, which is consistent with the treatment goal of DPN. Previously, we showed that WTHX-Cream can significantly improve clinical symptoms and signs, relieve pain, improve nerve conduction velocity, and delay DPN progression [4, 13]. The current study showed that WTHX-Cream can improve DPN and hyperalgesia symptoms, the mechanism of which may include regulation of PLC-*β*3 expression in the DRG.

In this study, we used a classic STZ injection method to establish diabetic rat models and assessed the DPN rat models for sciatic nerve conduction velocity after 8 weeks of continuous observation. The established DPN models showed the typical characteristics of DPN, such as elevated blood glucose, weight loss, and polyuria. A decreased pain threshold and increased pain sensitization are typical manifestations of DPN. After 4 weeks of WTHX-Cream treatment, the pain threshold of rats was increased, suggesting that WTHX-Cream can reduce pain by decreasing pain sensitization to improve the pain symptoms of DPN. However, no significant improvement in body weight or blood glucose was observed after 4 weeks of administration, and previous studies have shown that WTHX-Cream has no direct effect on blood glucose in DPN rats [14]. The possible reasons for the lack of effects were as follows: (1) the number of experimental animals was limited; (2) drug administration time was short, and drug plasma concentration was not fully maintained. However, WTHX-Cream had a definite effect on improving the pathological structure of DPN rats; for example, light microscopy showed that neuronal degeneration and nuclear fragmentation in the WTHX-Cream groups were significantly lower than those in the model groups; electron microscopy showed that the nerve myelin structure and vacuolar changes in WTHX-Cream groups were improved, and these improvements were more obvious with higher dosages of WTHX-Cream.

PLC-*β*3 is a subtype of the phospholipase C family and can bind to specific receptors on the cell membrane. Studies have shown that the PLC-*β*3 molecule in the rat DRG is a protein kinase C upstream molecule that leads to inflammatory pain. However, the mechanism underlying the pain caused by PLC-*β*3 is still unclear. The possible mechanisms are as follows: (1) PLC-*β*3 activation causes hydrolysis of phosphatidylinositol-4,5-diphosphate (PIP2), resulting in inositol triphosphate (IP3) and diacylglycerol (DAG) production. IP3 binds to the IP3 receptor on the endoplasmic reticulum and opens Ca^2+^ channels, thus promoting the release of intracellular stores of Ca^2+^ and increase of intracellular Ca^2+^. (2) PLC-*β*3 activation may cause the release of some irritating neurotransmitters, such as glutamate and irritant nerve polypeptide [15]. These neurotransmitters can cause pain, demonstrating that PLC-*β*3 has a pain-promoting effect. Local injection of the PLC-*β*3 inhibitor U73122 in mouse paws inhibited inflammatory hyperalgesia caused by a carrageenan injection at the same site [16], indicating that PLC-*β*3 can enhance pain sensitivity. In this study, the PLC-*β*3 mRNA or protein levels were significantly higher in the medium- and high-dose WTHX-Cream + metformin groups than in the model group at 4 weeks after using WTHX-Cream. The improvement was more obvious when the dosage was higher. Therefore, we hypothesized that WTHX-Cream can inhibit the decrease of PLC-*β*3 in DPN rats. Moreover, after drug application, the pain threshold of cold allodynia, heat pain, and mechanical pain was increased; nerve conduction velocity and sciatic nerve morphology were improved compared with those of the model group; and efficacy was better than that of the capsaicin cream + metformin group, indicating that WTHX-Cream may improve hyperalgesia of DPN by inhibiting downregulation of PLC-*β*3.

In WTHX-Cream, Chuanxiong not only promotes blood circulation to regulate the meridians but also promotes the operation of “qi” (the energy of life) to achieve an analgesic effect. Safflower is an important traditional Chinese medicine that promotes blood circulation and smooths the meridians. The combined use of the two herbs can increase blood circulation and dredging of the channel. Modern pharmacological studies have shown that Chuanxiong and its extracts have a direct expansion effect on peripheral blood vessels, and its effective component ligustrazine can dilate blood vessels, reduce platelet aggregation, reduce blood viscosity, reduce thrombosis, improve blood flow, and improve microcirculation effects [17]. Safflower contains ingredients such as carthamin and carthamin yellow, which can reduce blood viscosity, inhibit platelet aggregation, improve plasma fibrinolytic activity, prevent microthrombosis and dissolve thrombolysis, eliminate the effect on blood vessels caused by adrenaline and norepinephrine, and relieve smooth muscle spasm, thus leading to expansion of blood vessels, improvement in microcirculation [18], and improvement in neuronal ischemia and hypoxia. The herb “Guizhi” primarily contains components that include cinnamaldehyde, *β*-paclitaxel, and cinnamic acid, which result in pain relief and dilation of blood vessels.* Asarum* was first reported in “Shennong's Classic of Materia Medica” and was said to dispel wind, dissipate cold, move water, and relieve pain. Modern pharmacological studies have shown that* Asarum* has antipyretic, analgesic, and sedative effects [19].* Angelica* can also improve blood circulation in the lesion and relieve pain. In this study, the curative effect of WTHX-Cream was better than that found in the capsaicin cream + metformin group, indicating that WTHX-Cream does have a therapeutic effect on DPN. Furthermore, WTHX-Cream is an external medicine that avoids the first-pass effect of oral drugs into the body and reduces the damage to the gastrointestinal tract. Additionally, it directly acts on the lesion, is easy to use, has fast transdermal absorption, and is widely used in clinical practice. Modern research also shows that the external use of traditional Chinese medicine can hydrate the stratum corneum, which helps drug penetration and increases the drug transdermal rate by 4-5 times. Additionally, it increases skin temperature and hence accelerates blood circulation. WTHX-Cream added to the transdermal enhancer Azone can significantly promote transdermal absorption of most hydrophilic and hydrophobic compounds.

In summary, the multitarget role of traditional Chinese medicine in the treatment of DPN has been widely recognized, and an in-depth study of its mechanism will help elucidate the efficacy of Chinese medicine and thus provide new ideas for the treatment of diseases. In this study, we examined the inhibitory effect of WTHX-Cream on the downregulation of PLC-*β*3 at the gene and protein levels in the rat DRG and investigated the molecular mechanism of WTHX-Cream to reduce the sensory sensitivity of DPN, which is important for the clinical treatment of DPN. Our future studies will focus on the role and mechanism of the major components of WTHX-Cream.

## Figures and Tables

**Table 1:** PLC-*β*3 primer sequence.

Gene ID	Gene name	Primer sequence (5′ to 3′)	Product size (bp)
29322	Rat PLC-*β*3	F: CAAGCCGTACCTCACTCTGG	169
	Rat PLC-*β*3	R: CATAGACATCTGGTCGCGCT	
